# Supplementary material for: Inhibitory Effect of Fucoidan Analogs on Highly Metastatic Gastric Cancer Cells via Galectin-4 Inhibition
Source: Int J Mol Sci. 2025 Sep 21;26(18):9228. doi: 10.3390/ijms26189228 (PMC12470716; doi:10.3390/ijms26189228)
Supplement: Supplementary file 1 [file ijms-26-09228-s001.zip › ijms-3727862-supplementary.pdf]

Supporting information

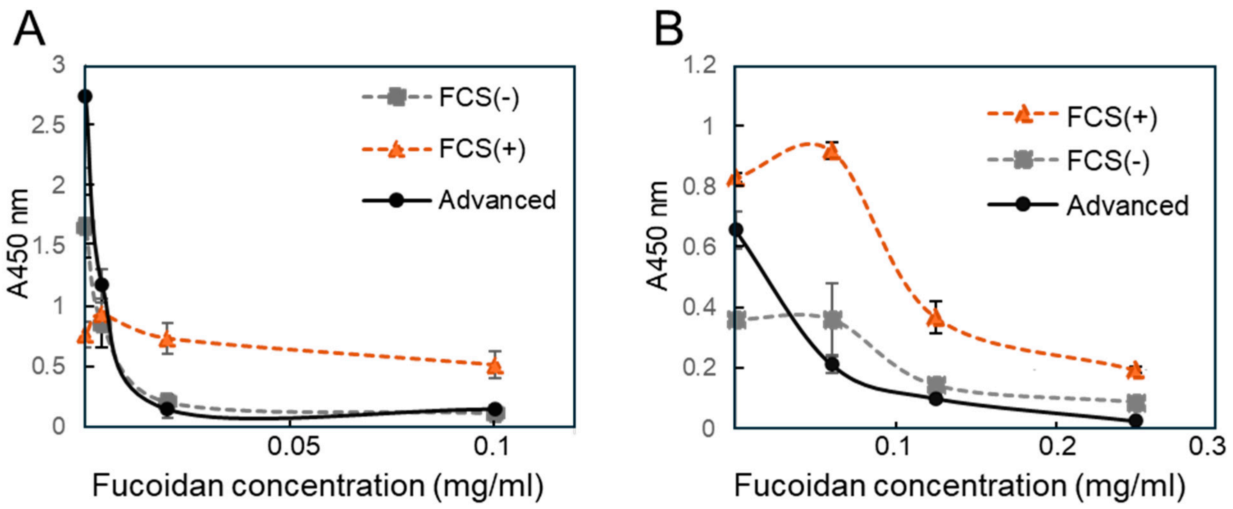

Figure S1. Effect of serum on the inhibitory activity of natural fucoidan. (A) Inhibitory activity of fucoidan on galectin-4 binding. Natural fucoidan solution was diluted in RPMI 1640 medium with or without 10% FCS or Advanced RPMI 1640 medium. They were then added to each well of a cholesterol 3-sulfate-coated plate. (B) Inhibitory activity of natural fucoidan on cell proliferation. Natural fucoidan solution was diluted in RPMI 1640 with or without 10% FCS, or Advanced RPMI 1640 medium. They were added to the cells and cultured for another 3 days. FCS, fetal calf serum.

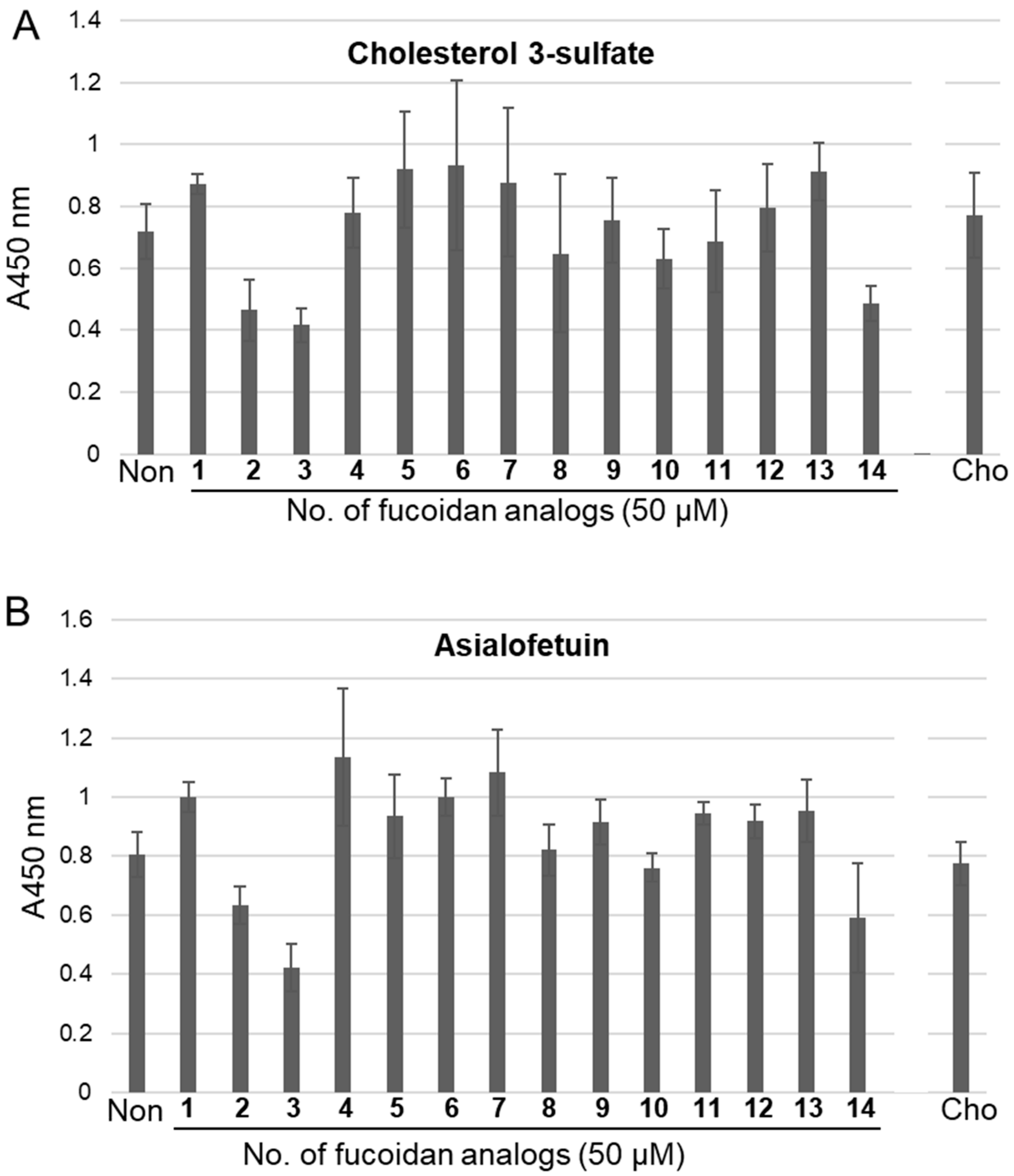

Figure S2. Inhibitory activity of fucoidan analogs on galectin-4 binding. The fucoidan analogs (50 μM) were incubated with galectin-4 and applied to (A) cholesterol 3-sulfate (B) asialofetuin-coated plate. Non, non-treated; Cho, cholestanol

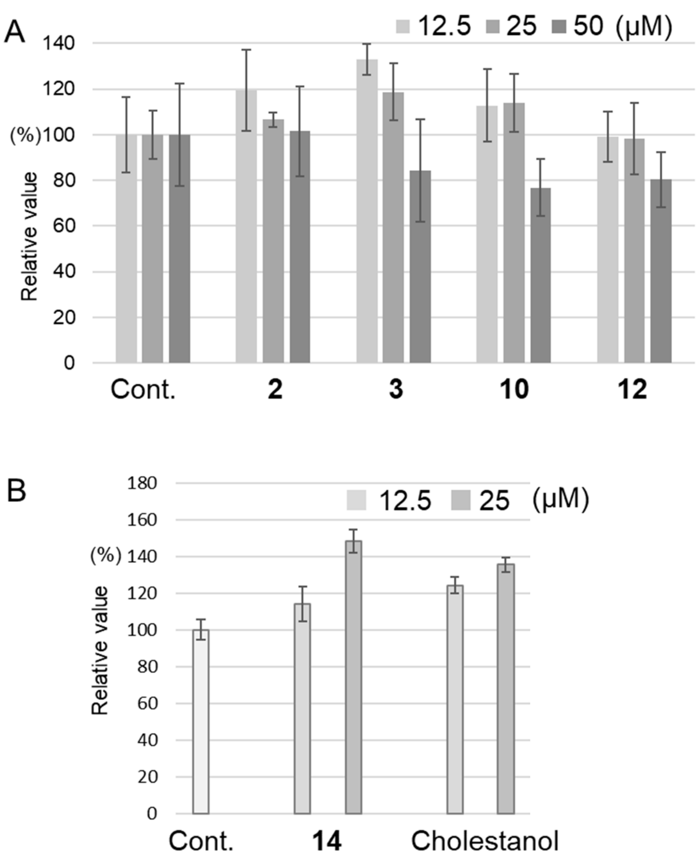

Figure S3. The effect of fucoidan analogs on the proliferation of HEK293 cells. (A) The effect of fucoidan analogs **2**, **3**, **10**, **12**, (B) **14** and cholestanol on the proliferation of HEK293 cells. Cell proliferation was determined using an ATP assay at 72 h after treatment. The value from the ATP assay of untreated cells was set to 100%, and the relative values of the treated cells are presented. Cont., control

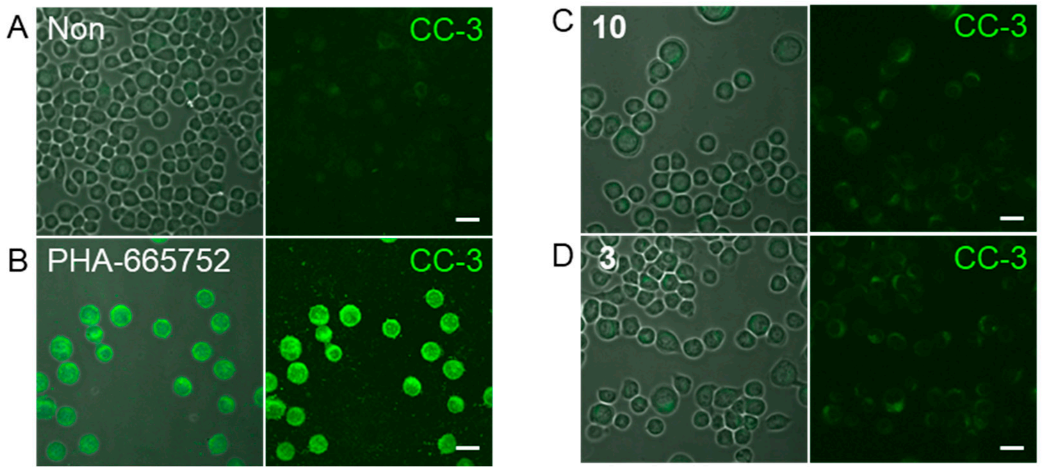

Figure S4. Immunofluorescence labeling of the cleaved-caspase-3. NUGC4 cells were incubated with (A) advanced RPMI 1640 medium alone, (B) 2 µM PHA-665752, (C) 50 µM **10**, and (D) 50 µM **3** for three days. Stained images with anti-cleaved-caspase-3 and the merged confocal images with corresponding differential interference are shown (Scale bar, 20 µm). CC-3, cleaved-caspase-3.

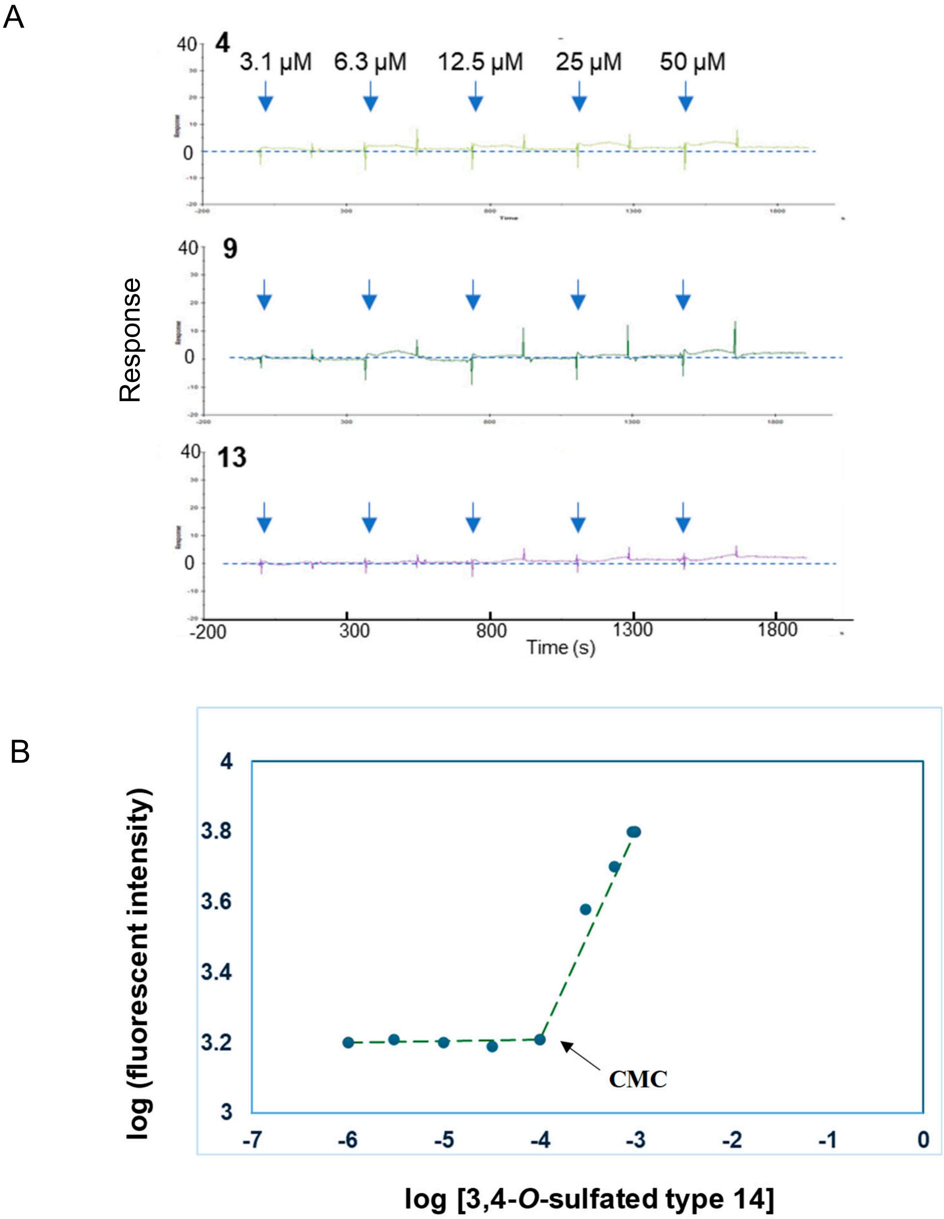

Figure S5. (A) SPR sensorgrams for the binding of fucoidan analogs to galectin-4. Increasing concentrations of **4**, **9**, and **13** were introduced into galectin-4 immobilized on the surface. The relative response was determined by subtracting the blank values obtained for the non-immobilized surface from those obtained for the galectin-4-immobilized surface.

(B) Determination of the CMC of **14** with 1 µM NPN in HBS-EP buffer. NPN fluorescence emission intensity as a function of the concentration of **14**. The intersection of the two straight lines indicates CMC.

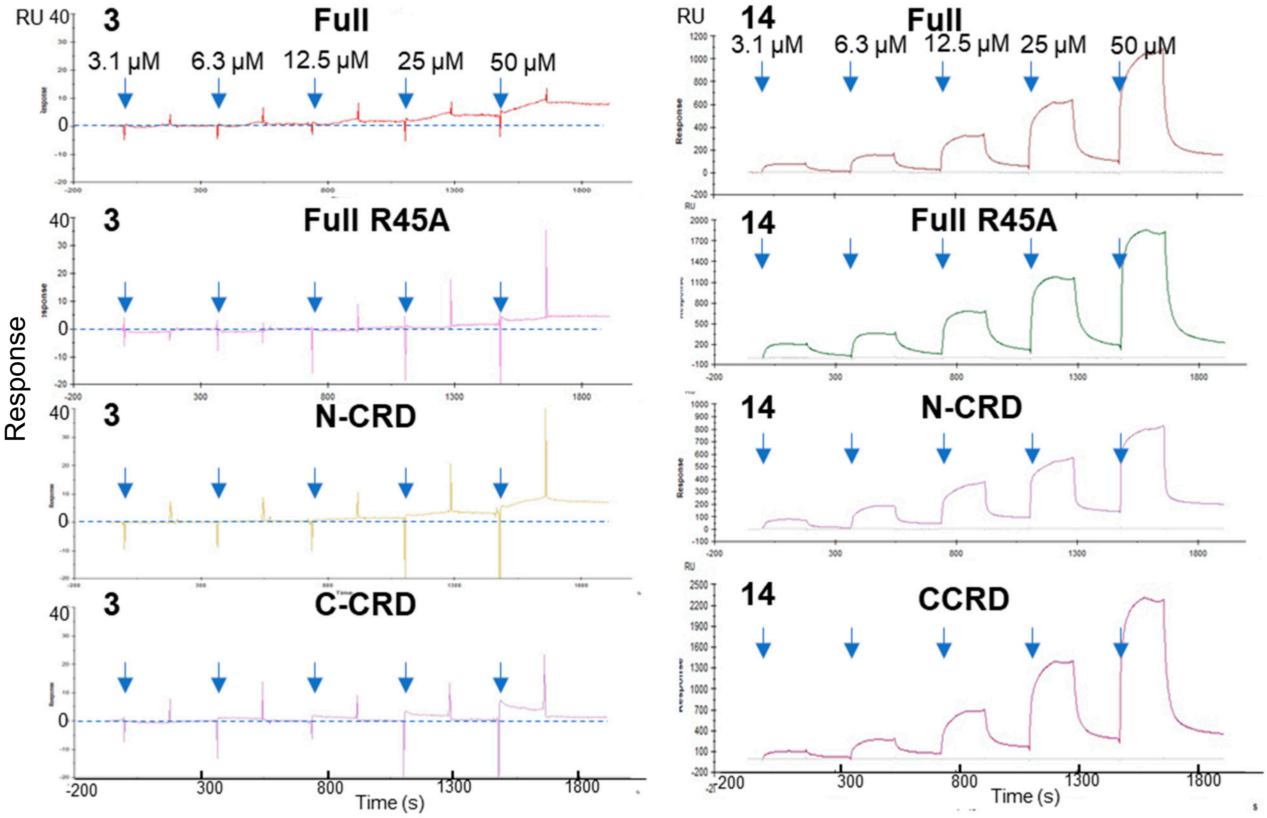

Figure S6. SPR sensorgrams for the binding of fucoidan analogs to galectin-4. Increasing concentrations of fucoidan analogs **3** and **14** were introduced into full-length galectin-4 WT, mutant R45A, N-CRD, and C-CRD immobilized on the surface. The relative response (RU) was determined by subtracting the blank values obtained for the non-immobilized surface from those obtained for the galectin-4-immobilized surface. CRD, carbohydrate-recognition domain; SPR, surface plasmon resonance

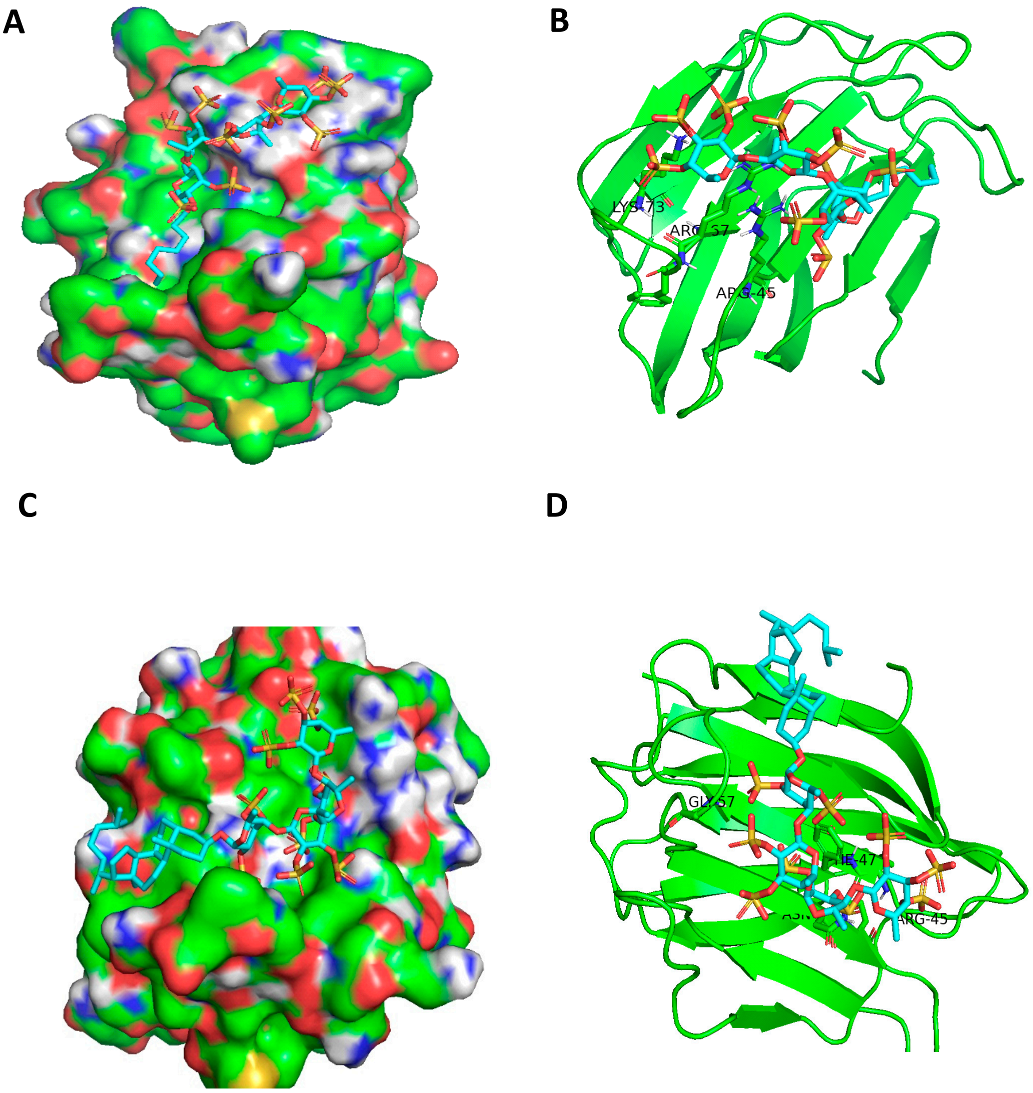

Figure S7. Molecular docking simulation. (**A**) 3D Molecular surface representation of 5DUW-**10** complex, 5DUW surface is color-coded based on atom types: green for carbon, red for oxygen, blue for nitrogen, and white for hydrogen. The ligand **10** is displayed in a stick model with carbon (cyan), oxygen (red), and sulfur (yellow) atoms. (**B**) 5DUW is shown in green as a ribbon diagram, highlighting β-sheet structures. The ligand **10** is depicted in stick representation with carbon (cyan), oxygen (red), and sulfur (yellow) atoms. Key interacting residues, including Arg45, Arg67, and Lys73 are properly labeled and represented in stick format. (**C**) 3D Molecular surface representation of 5DUW-**14** complex, 5DUW surface is color-coded based on atom types: green for carbon, red for oxygen, blue for nitrogen, and white for hydrogen. **14** is displayed in a stick model with carbon (cyan), oxygen (red) and sulfur (yellow) atoms. (**D**) 5DUW is shown in green as a ribbon diagram, highlighting β-sheet structures. The ligand **14** is depicted in stick representation with carbon (cyan), oxygen (red) and sulfur (yellow) atoms. Key interacting residues, including Arg45, Phe47, Gly57, and Asn65 are properly labeled and represented in stick format. Visualization of the images were done with PyMOL.

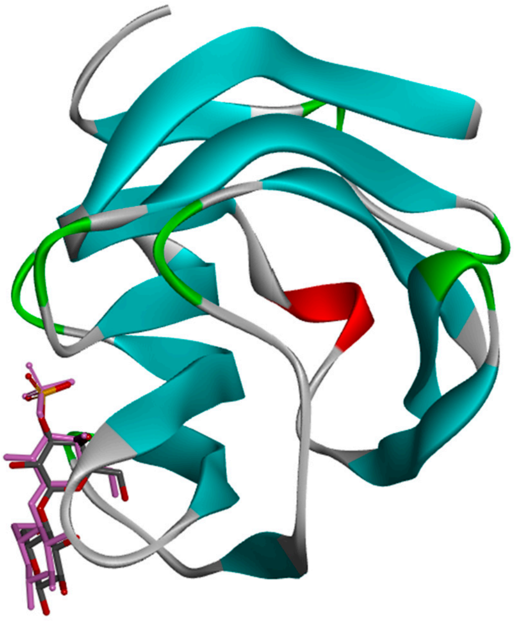

Figure S8. Redocking of the co-crystallized ligand, lactose-3′-sulfate into the binding site of 5DUW. The crystal structure of the monomeric chain of 5DUW is shown as a ribbon diagram (β-strands in cyan, α-helices in red, and loops in grey/green). The co-crystallized native ligand, lactose-3′-sulfate, is shown in its original position, while the redocked pose is highlighted in pink.

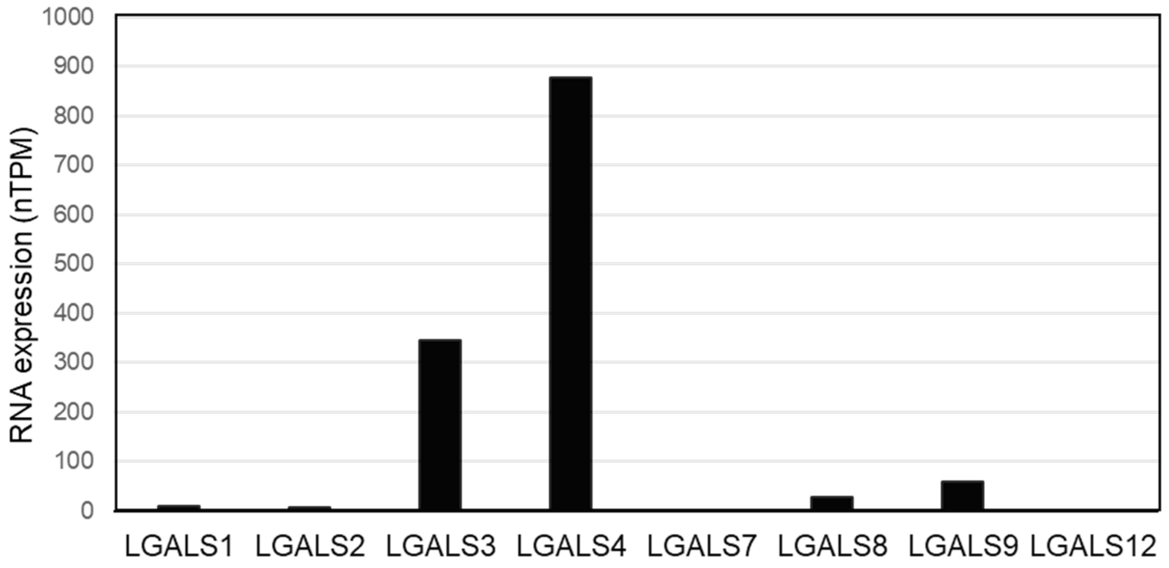

Figure S9. RNA expression of galectins in NUGC4 cells. RNA expression data as normalized transcript per million (nTPM) values of NUGC4 cells are graphed. LGALS5, LGALS6, LGALS5, LGALS10, and LGALS11 are expressed in other species. We obtained nTPM data for human galectin genes from the Human Protein Atlas (HPA) database (https://www.proteinatlas.org/ (accessed on 12 August 2025)) [47. Uhlén, M.; Fagerberg, L.; Hallström, B.M.; Lindskog, C.; Oksvold, P.; Mardinoglu, A.; Sivertsson, Å.; Kampf, C.; Sjöstedt, E.; Asplund, A.; et al. Proteomics. Tissue-Based Map of the Human Proteome. Science 2015, 347, 1260419.] https://doi.org/10.1126/science.1260419.].

Table S1. Summary table of structures and inhibitory effects of synthesized fucoidan analogs.

	Fucose linkage	MW	N. of SO_3	Sulfated pattern	Growth inhibition (IC50)		Binding inhibition (IC50)[a]
					MKN45	NUGC4 WT	
**1**	α(1,3)-	1633	9	2,4-O-	>100 μM	–[b]	–
**2**	α(1,3)-	1225	5	4-O-	>100 μM	> 50 μM	>100 μM
**3**	α(1,3)-	1225	5	2-O-	>100 μM	> 50 μM	20~100 μM
**4**	α(1,3)-	715	0	Non	>100 μM	–	–
**5**	α(1,3)- & α(1,4)-	1633	9	2,3,4-O-	>100 μM	–	–
**6**	α(1,3)- & α(1,4)-	1429	7	2,3-O-	>100 μM	–	–
**7**	α(1,3)- & α(1,4)-	1225	5	3,4-O-	>100 μM	–	–
**8**	α(1,3)- & α(1,4)-	1021	3	4-O-	>100 μM	–	–
**9**	α(1,3)- & α(1,4)-	715	0	Non	>100 μM	–	–
**10**	α(1,4)-	1633	9	2,3-O-	≃100 μM	< 50 μM	20~100 μM
**11**	α(1,4)-	1225	5	3-O-	>100 μM	–	–
**12**	α(1,4)-	1123	4	2-O-	>100 μM	> 50 μM	20~100 μM
**13**	α(1,4)-	715	0	Non	>100 μM	–	–
**14**	α(1,4)-	1892	9	2,3-O-	12.5~25 μM	< 20 μM	< 4 μM
**14** is the cholestanol-conjugated form of **10**.							

MW, molecular weight; N. of SO_3, number of sulfate groups per molecule.

[a]: The inhibitory activity of fucoidan analogs against galectin-4 binding to blood group A type 1-neoglycolipid.

–[b]: not tested.
